# Current Status of Genetic Diagnosis Laboratories and Frequency of Genetic Variants Associated with Cystic Fibrosis through a Newborn-Screening Program in Turkey

**DOI:** 10.3390/genes12020206

**Published:** 2021-01-31

**Authors:** Sevcan Tug Bozdogan, Cem Mujde, Ibrahim Boga, Ozge Sonmezler, Abdullah Hanta, Cagla Rencuzogullari, Dilek Ozcan, Derya Ufuk Altintas, Atil Bisgin

**Affiliations:** 1 Medical Genetics Department, Faculty of Medicine, Cukurova University, Adana 01250, Turkey; sevcantb@gmail.com (S.T.B.); ibr.boga@gmail.com (I.B.); 2 AGENTEM (Adana Genetic Diseases Diagnosis and Treatment Center), Cukurova University, Adana 01250, Turkey; cemmujde@gmail.com (C.M.); ozgesonmezler@gmail.com (O.S.); abdullahhanta@gmail.com (A.H.); caglaorucrencuz@gmail.com (C.R.); 3 Faculty of Medicine, Department of Pediatric Allergy and Immunology Adana, Cukurova University, Adana 01250, Turkey; dilekkaragoz1977@hotmail.com (D.O.); deryaufuk@gmail.com (D.U.A.)

**Keywords:** cystic fibrosis, newborn screening, genetic testing, population genetics

## Abstract

Background: Cystic fibrosis (CF) is the most common worldwide, life-shortening multisystem hereditary disease, with an autosomal recessive inheritance pattern caused by mutations in the cystic fibrosis transmembrane conductance regulator (*CFTR*) gene. The national newborn screening (NBS) program for CF has been initiated in Turkey since 2015. If the immunoreactive trypsinogen (IRT) is elevated (higher than 70 μg/L in the second control) and confirmed by sweat test or clinical findings, genetic testing is performed. The aims of this study are to emphasize the effect of NBS on the status of genetic diagnosis centers with the increasing numbers of molecular testing methods, and to determine the numbers and types of *CFTR* mutations in Turkey. Methods: The next-generation sequencing (NGS) and multiplex ligation-dependent probe amplification (MLPA) results of 1595 newborns, who were referred to Cukurova University Adana Genetic Diseases Diagnosis and Treatment Center (AGENTEM) for molecular genetic testing, were evaluated with positive CF NBS program results since 2017. Results: According to the results; 560 (35.1%) of the 1595 patients carried at least 1 (one) CF-related variant, while 1035 patients (64.9%) had no mutation. Compound heterozygosity for two mutations was the most common in patients, while two detected variants were homozygote in 14 patients. A total of 161 variants were detected in 561 patients with mutations. Fifteen novel variants that have not been previously reported were found. Moreover, p.L997F was identified as the most frequent pathogenic mutation that might affect the IRT measurements used for the NBS. The distribution of mutation frequencies in our study showed a difference from those previously reported; for example, the well-known p.F508del was the third most common (*n* = 42 alleles), rather than the first. The most striking finding is that 313 cases had a pathogenic variant together with the V470M variant, which might have a cumulative effect on CF perpetuation. Conclusion: This study is the first to determine the mutational spectrum of CFTR in correlation with the NBS program in the Turkish population. NBS for CF raises issues regarding screening in diverse populations, both medical and non-medical benefits, and carrier identification. Through the lens of NBS, we focused on the integrated diagnostic algorithms and their effect on the results of genetic testing.

## 1. Introduction

Cystic fibrosis (CF) is the most common worldwide, life-shortening multisystem disease with an autosomal recessive inheritance pattern affecting 1 in 3300 to 1 in 4800 neonates and 1 in 2500 white individuals [1,2]. CF is a hereditary disease (MIM #219700) caused by mutations in the cystic fibrosis transmembrane conductance regulator (*CFTR*) gene (MIM *602421) [3]. The cystic fibrosis gene, *CFTR*, was firstly identified in 1989, is located at the 7q13 position [4], and spans ~190 kilobases (kb) on chromosome 7q31.2 with 27 exons. The *CFTR* gene encodes the *CFTR* protein that functions as a Cl-selective anion channel gated by cycles of ATP binding and hydrolysis at its nucleotide-binding domains [5]. Regarding *CFTR* gene mutations, to date, there are 1908 identified mutations, of which 982 are missense or nonsense [6], while the deletions and duplications of complete exons account for 1–2% of all mutations [7].

Mutations in the *CFTR* gene may result in defective protein processing that leads to changes in function and regulation of the chloride channel. CF affects exocrine glands; chloride secretion is diminished, sodium absorption and removal of water from secretions are increased through epithelial sodium channels, and the secretions are therefore abnormally viscous [8]. It mainly involves the lungs and pancreas, but also the upper airways, liver, intestine, and reproductive organs; 99% of the affected male patients are infertile due to obstructive azoospermia, and 87% of patients have exocrine pancreatic insufficiency. Its importance is due to the fact that it is a life-shortening disease, and the median predicted survival age and the median life expectancy are 47.4 and 44.4 years, respectively [9]. The longer patients with CF live, the worse their quality of life may become, in addition to the increased burden and cost of treatment [10]. Morbidity and mortality in CF are attributed most commonly to pulmonary disease, characterized by chronic lung infections and airway inflammation. Other common clinical manifestations are failure to thrive, pancreatic insufficiency, meconium ileus, and infertility resulting from a congenital bilateral absence of the vas deferens [1].

The national newborn screening (NBS) program for CF in Turkey has been in operation since 2015, and it was initiated by the Public Health Institution of Turkey. Immunoreactive trypsinogen (IRT) measurement as a first-tier testing method for newborns is used in NBS. Infants who are identified as positive in the NBS program are directed to CF centers for sweat testing followed by clinical assessment by pediatric allergy and immunology specialists. Then, the infants are referred to medical geneticists and genetic diagnosis centers for molecular genetic testing to also identify the mutational status and provide counseling to the family. Even though the screening tests have low false-positive rates, sweat testing and careful physical examination by pediatric immunologists are performed. As of 2017, genetic analyses via next-generation sequencing have been carried out at the main core center.

With this new NBS program, Turkey has taken a giant leap, screening a total of 1.2 million live births in 2019 and 1.25 million in the previous year according to the country’s statistical institute (TUIK) [11]. For this NBS, the entire program’s goal is to identify the disease and provide clinical answers to newborns. However, when screening via the IRT protocol, genetic diagnosis centers were required to overcome the large testing numbers and examine the data to identify connections with disease and variants, as research in this area is lacking in the Turkish population (excluding a single study from the IRT side with limited and early data [12]).

As a core center in our region, serving an area from south-east to the south-west of Turkey and dealing with all genetic diseases as well as CF molecular testing since its establishment, our aim within this study is, therefore, to identify how an NBS program for CF can affect the genetic diagnosis center’s status with the increasing numbers of molecular testing methods, from the used techniques to the genotype variation of the *CFTR* gene. Moreover, in this study, we used the population-based globally well-known datasets, such as gnomAD and CFTR2, in order to assist genetic diagnostic laboratories and to demonstrate the analysis of large cross-population sequencing data in a large cohort that can significantly improve disease variant interpretation by assessing the variant frequency for our population with the varying prevalence.

## 2. Materials and Methods

### 2.1. Patients and Sampling

Peripheral blood samples of 1595 newborns with positive CF NBS program results since April 2017 who were to Cukurova University Adana Genetic Diseases Diagnosis and Treatment Center (AGENTEM) referred for molecular genetic testing were included in this study. The CF newborn screening protocol in Turkey includes several steps, starting from sampling blood samples of newborns via sampling cards (Guthrie cards) at 72 h of life. Those whose first IRT level is higher than ≥90 μg/L were called for a second IRT measurement 7–14 days after birth. Then, if it is above ≥70 μg/L, the infant was directed to the nearest CF center. The referred patients were evaluated at least once per three months, and the sweat test was performed for each infant with a gestational age of 38 weeks or more and a minimum weight of 2000 g together with clinical assessment. The NBS program’s CF sweat testing results are considered to be abnormal when they are greater than 90 mmol/L.

Informed parental consent was obtained for all patients in accordance with the ethical standards of the institutional ethical committee (Cukurova University Faculty of Medicine Non-Invasive Clinical Research Ethics Commission) and the Helsinki declaration.

Peripheral blood samples were collected for genomic DNA isolation via the QIAamp DNA Blood Mini Kit (Qiagen, Hilden, Germany), according to the manufacturer’s instructions. The quality of DNA samples was assessed with a Qubit Fluorimeter (Thermo Fisher Scientific, Waltham, MA, USA).

### 2.2. Next-Generation Sequencing (NGS)

The next-generation sequencing workflow was performed to achieve a minimum of 300X coverage on an Illumina MiSeq (Foster City, CA, USA) platform via a custom-designed by our center’s *CFTR* gene panel (QIAseq Targeted DNA Custom Panels—CDHS-12025Z-65, QIAgen, Hilden, Germany), including all exons, introns, and 1 kb of the 5′ promoter regions and the 3′ UTRs.

### 2.3. Bioinformatics Analyses

Quality control parameters were checked for both sequencing and variant qualities via the QIAGEN Clinical Insight (QCI) Analyze tool and the QCI Interpret interface. Total yield, sequencing quality score, depth of coverage, the quality score of variants, forward/reverse read balance, population, and variant frequencies were assessed as primary variant analysis. Variants were categorized based on their pathogenicity according to the American College of Medical Genetics (ACMG) criteria as pathogenic, likely pathogenic, variant of uncertain significance (VUS), likely benign, and benign. In silico analysis tools, including SIFT, B-SIFT, Polyphen-2, MutationTaster, BLOSUM, PROVEAN, CADD, DANN, GeneSplicer, PhyloP, MaxEntScan, and QCI Inferred Activation, were also used for the further examination of the VUSs.

### 2.4. Multiplex Ligation-Dependent Probe Amplification (MLPA) Analyses

All of the samples that were negative or had a heterozygote mutation for *CFTR* next-generation sequencing were then screened to identify the deletions of the *CFTR* gene via MLPA (*CFTR*-SALSA-MLPA-P091, MRC-Holland, Amsterdam, Netherlands) following the manufacturer’s recommendations and protocol on ABI 3130XL (Applied Biosystems, Foster City, CA, USA).

The sizes of the exon-specific amplified fragments were identified according to their migration relative to the GeneScan Rox-500 size standard (Applied Biosystems, Foster City, CA, USA) using GeneMapper version 4.0 software. The relative copy numbers of the *CFTR* gene products were determined using Coffalyser software provided online by the manufacturer (www.mlpa.com). We considered results with values between 0.8 and 1.2 as normal. The relative copy number values above 1.3 were considered duplications, and values below 0.65 were considered deletions.

## 3. Results

NBS for CF in Turkey has also introduced widespread DNA-based testing for patients. NGS has become the preferred molecular genetics testing method due to its high sensitivity and reliability. It enables multigene sequencing of multiple samples during the same workflow. This provides copious amounts of data and information as compared to conventional methods. This technology, however, requires qualified laboratory scientists and experienced medical geneticists to process and analyze such a large quantity of data.

According to the results, 560 (35.1%) of the 1595 patients carried at least 1 (one) homozygous or heterozygous CF-related variant, while 1035 patients (64.9%) had no detected clinically significant variants.

**Figure 1:**
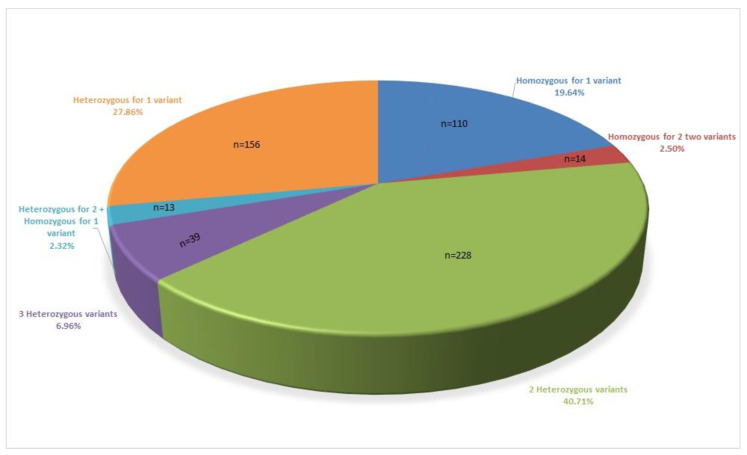
Distribution of patients according to mutation burden.

Among the patients who had at least one disease-related variant, 110 had a homozygous mutation for at least one variant (110/560 = 19.64%), and 228 patients had two different variants in the compound heterozygous state (40.71%). Fifty-two of the patients had three variants (9.3%); 39 of these 52 had three different mutations in the heterozygous state; 13 patients were homozygous for one mutation and heterozygous for two mutations. One hundred fifty-six (156) patients had one heterozygous mutation (27.86%); 14 patients had double homozygous mutations (homozygous for two different mutations simultaneously; 2.5%; Figure 1).

We detected 15 novel variants that have not been previously reported (Table 1). We found a total of 161 variants in 560 patients with mutations (Table 2).

**Figure 2:**
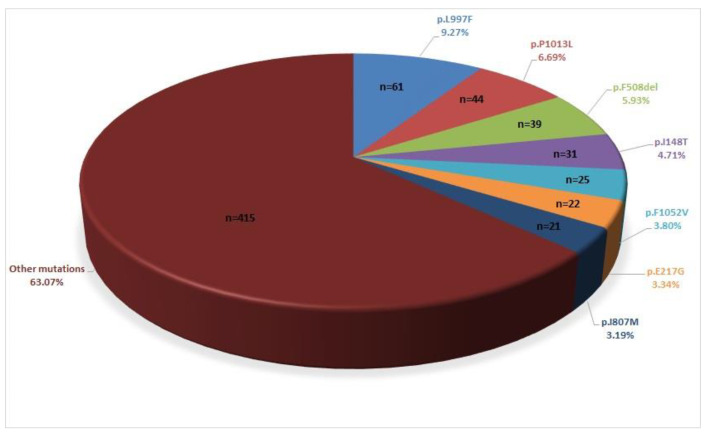
Distribution of the most frequent mutations.

The most frequent variant was p.V470M; 313 of 560 patients had this variant. p.V470M is classified as a polymorphism according to ACMG, and it is known to have a cumulative effect when combined with other mutations [13]. Therefore, this mutation was reported only when the patient had other clinically significant mutations (figures were prepared excluding p.V470M). Thus, no other common or benign variants were reported other than p.V470M. The most frequent pathogenic mutation, p.L997F, was detected in 61 of 560 patients. The second most frequent pathogenic variant was p.P1013L, and it was detected in 44 of 560 patients. p.F508del was the third most frequent mutation, observed in 39 of 560 patients (Figure 2).

**Figure 3:**
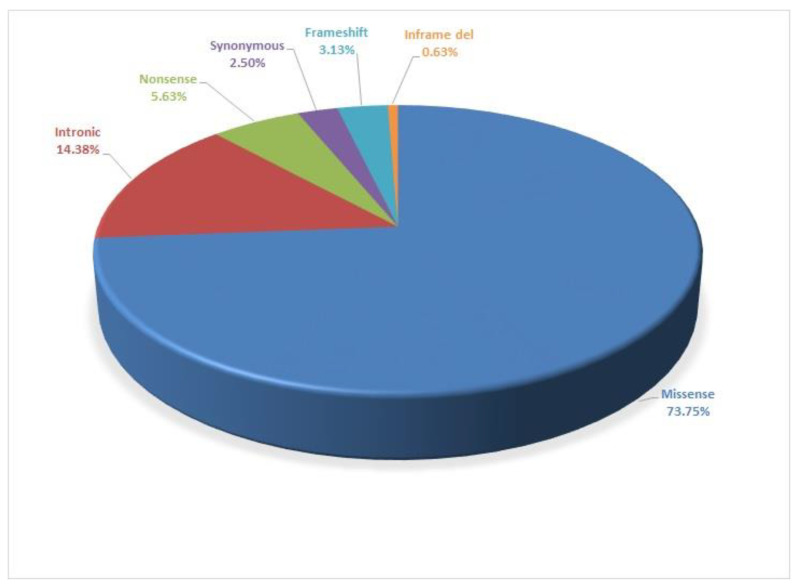
Description of the detected types of mutations.

The vast majority of the mutant alleles were single base-pair substitutions; 118 of the 160 variants were missense mutations. Twenty-three of the patients had an intronic variant, while nine patients had a nonsense mutation (calculations were made excluding p.V470M; Figure 3).

Deletion–duplication of *CFTR* was investigated with the MLPA method in 391 patients with no mutations, and deletion was detected in 3 patients. Two of three patients had exon 10 deletions, and the others had multiple exon (exon 4–11) deletion—these patients had no mutations detected by sequencing (MLPA results are not shown in tables and figures).

## 4. Discussion

Newborn genetic screening has been a remarkable achievement as a public health intervention, providing population-wide detection of disorders that have greatly improved the lives of thousands of affected children and even more than a million newborns each year. However, both the economic and social forces pose significant ethical and clinical challenges to NBS, and they mainly regard accommodating laboratory and clinical standards to rapid developments and preparing health systems to respond to such advances.

With the trend of increasing molecular test requests, all genetic testing laboratories have been forced to adapt to the times and utilize technology with high sensitivity and reliability. As a result of the CF NBS program, due to the high number of samples that require quick and trustworthy results, next-generation sequencing has become a first-tier testing method, largely replacing targeted sequencing or other conventional methodologies. However, even with powerful test technologies such as NGS, there remains a need to multiplex such techniques, as conducted in this study with the combination of NGS and MLPA used to identify newborns or children with anomalies that may—or may not—lead to disease. Furthermore, if these NBS programs evolve and include more than a single disease testing method, they may also be able to find children for whom treatment is available.

In regard to CF, more than 2000 mutations have been identified in the *CFTR* gene, which may lead to a loss of function of this anion channel at the apical plasma membrane of secretory epithelia [14]. The majority of all of these variants are point mutations or other small sequence changes, and up to 2% of CF alleles are likely gene rearrangements, including large deletions, insertions, and duplications [15]. However, we detected large deletions in only 3 patients (two heterozygous and one homozygous, a total of four deleted alleles) among the 391 patients on whom MLPA analysis was conducted. This accounts for 0.5% of 391 patients (782 alleles), which is a lower figure than that in published cohort studies.

The most common *CFTR* mutation, p.F508del (delF508 by legacy nomenclature), accounts for approximately 66–70% of identified mutant alleles worldwide [16,17] while it was observed in only 39 (6%) patients with at least one mutation, making it the third most frequent mutation in our study. Even though the p.F508del mutation accounts for 70% of CF mutations in white patients of northern European descent, it has differing percentages in other populations [18]. In the Russian population, the p.F508del mutation accounts for 53% of mutations [19], and this is caused by the specific ethnic background of the population. Thus, screening for known CF gene mutations did not help to ameliorate such inequities in our population, but the combination of NGS with MLPA was more successful, as explained in the Results Section. NGS has dramatically advanced the process of genetic variant identification; however, clinical interpretation remains a challenge. Even though the largest publicly-available population datasets for CF to date are gnomAD and CFTR2, participants are classified into seven different ethnic groups without clustering any other major populations, which are most likely of mixed backgrounds, especially in Mediterranean countries.

The most frequent mutation in the Turkish population according to this study, in which all of the patients enrolled were selected by the NBS program, is p.L997F. Thus, this study provides important data to clarify the complexity of this variant in relation to the disease [20]. L997F was identified by Lucarelli et.al. who reported that it leads to mild CF or cystic fibrosis-related disorders (CFRD) [21]. In other reports, this variant was associated with pancreatitis [22,23], CFRD, and mild CF [24]. There are publications stating that this variant has no relation with CF disease, and there are also publications opposing the notion that it is causative [21,25]. Since the false-negative rate of NBS is at least 8.7%, depending on the method, patients with this mutation who receive false negative NBS results have an increased risk of presentation with meconium ileus after birth or dehydration and pancreatitis at a later age [26].

Similarly, p.I148T is classified as VUS according to the ACMG criteria, with publicly open web-based datasets from VARSOME showing evidence-based data of its relation to CF. Moreover, several studies also suggest that it might have a cumulative effect on CF and/or the CFRD phenotype with congenital agenesis of vas deferens perpetuation when identified with other well-known pathogenic variants in different populations [24,27,28].

In contrast with the results of our study, it has been suggested in some studies that while the effect of the V470M variant is classified as benign according to the ACMG criteria, it is clinically significant when it is combined with other pathogenic variants [13,29]. Thus, it is only shown in Table 2, displaying the variant distribution. There is also a possibility that some variants such as V470M at a higher frequency may act as disease modifiers rather than casual variants; therefore, it is important for phenotype interpretation. There is also a chance that the CF genotype prevalence of V470M in gnomAD might be inflated since the analyses were allele based. However, this requires further investigations involving deep phenotyping and prospective phenotype correlation analysis, which our center intends to conduct in future studies.

In recent years, a considerable effort was focused on molecular therapies that can directly interact with *CFTR* mutants has indicated the importance of identifying the mutations of *CFTR* and the complexity of *CFTR* mutant phenotypes at the cellular levels [10,30,31]. Moreover, early diagnosis through NBS is the best way to prevent primary and secondary manifestations of the disease.

In populations such as those in the Mediterranean region, consanguineous marriage makes the incidence of autosomal recessive diseases such as CF in this study higher. Most interestingly, we reported double homozygous *CFTR* mutation in transposition status among patients whose parents were first-degree cousins, and their clinical picture was severe. Double homozygous *CFTR* mutations are a very rare phenomenon, but they are reported more commonly in the Saudi population due to consanguinity [32]. Thus, more precautions and family counseling to increase awareness about the risk of such relationships should be conducted to prevent such extremely rare phenomena. Genetic counseling and medical genetic assessment, together with the diagnostic approach, are becoming increasingly important. Advances in medical genetics and testing technology permit the diagnosis of ever more diseases but also compel society to reconsider how NBS as a public health measure may best serve children, their families, and their communities.

NBS for CF as a public health program in Turkey has achieved enormous success in regard to genetic testing to the extent that it may be implemented in other areas. Moreover, the most crucial step for effective CF management is the early and accurate diagnosis, as provided by this program, and our datasets are available to other centers for variant interpretation.

## Figures and Tables

**Table 1:** Detected novel variants and American College of Medical Genetics (ACMG) criteria provided with pathogenicity classification.

Variants	Mutation Type	ACMG Classification	Patient Numbers
c.2978A > C p.D993A	Missense	LP	7
c.1729T > C p.Y577H	Missense	VUS	2
c.3468 + 52A > C	Intronic	VUS	2
c.3170C > G p.T1057R	Missense	LP	1
c.2982C > G p.F994L	Missense	VUS	1
c.1680 − 756C > T	Intronic	VUS	1
c.2054A > G p.Q685R	Missense	LP	1
c.698T > C p.L233P	Missense	LP	1
c.4321delC p.R1438fs*10	Frameshift	LP	1
c.3468 + 137T > C	Intronic	VUS	1
c.7A > T p.R3W	Missense	VUS	1
c.1836A > T p.K612N	Missense	LP	1
c.1217G > C p.G406A	Missense	VUS	1
c.53 + 28A > G	Intronic	VUS	1
c.3311A > G p.E1104G	Missense	LP	1

LP: Likely Pathogenic, VUS: Variant of Uncertain Significance.

**Table 2:** The total list of mutations detected. * Not expected to have a major effect on the disease but have a cumulative effect with other pathogenic variants.

Variants	Mutation Type	Number of Alleles	ACMG Classification	CFRT2 Database
p.V470M	Missense	399	Benign *	N/A
p.L997F	Missense	66	P	Non CF-causing
p.P1013L	Missense	47	P	N/A
p.F508del	Inframe del	42	P	CF-causing
p.I148T	Missense	31	VUS	Non CF-causing
p.F1052V	Missense	25	P	Varying clinical consequence
p.E217G	Missense	22	P	N/A
p.I807M	Missense	22	P	Non CF-causing
c.2657 + 5G > A	Intronic	19	P	CF-causing
c.2620 − 15C > G	Intronic	16	P	N/A
c.3964 − 3C > G	Intronic	16	P	N/A
p.F1052L	Missense	13	P	N/A
p.K68E	Missense	13	P	N/A
p.D1312G	Missense	11	P	N/A
p.D993A	Missense	9	LP	N/A
p.M952I	Missense	9	P	N/A
p.Y515 *	Nonsense	9	P	N/A
c.2909 − 71G > C	Intronic	8	P	N/A
c.3718 − 2477C > T	Intronic	8	P	CF-causing
p.D110H	Missense	8	P	CF-causing
p.D1152H	Missense	8	P	Varying clinical consequence
p.K684fs*38	Frameshift	8	P	N/A
p.M348K	Missense	8	P	N/A
p.W1282 *	Nonsense	8	P	CF-causing
p.D806G	Missense	7	P	N/A
p.G576A	Missense	7	VUS	Non CF-causing
p.L732 *	Nonsense	7	P	CF-causing
p.N1303K	Missense	7	P	CF-causing
p.R347P	Missense	7	P	CF-causing
p.I506V	Missense	6	VUS	N/A
p.L633I	Missense	6	P	N/A
p.R117C	Missense	6	P	CF-causing
p.T388M	Missense	6	P	N/A
c.489 + 3A > G	Intronic	5	P	Varying clinical consequence
p.G542 *	Nonsense	5	P	Varying clinical consequence
p.R668C	Missense	5	P	Non CF-causing
p.R75Q	Missense	5	P	Non CF-causing
p.S1235R	Missense	5	P	Non CF-causing
p.S877A	Missense	5	VUS	N/A
p.T1220I	Missense	5	VUS	N/A
p.D513G	Missense	4	P	CF-causing
p.L1034F	Missense	4	P	N/A
p.P111L	Missense	4	P	N/A
p.Q493P	Missense	4	P	N/A
p.R117H	Missense	4	P	Varying clinical consequence
p.R297Q	Missense	4	P	N/A
p.W1098C	Missense	4	VUS	CF-causing
p.A120T	Missense	3	LP	Varying clinical consequence
p.A399V	Missense	3	P	N/A
p.D836Y	Missense	3	P	Non CF-causing
p.E1228G	Missense	3	LP	N/A
p.E528K	Missense	3	LP	N/A
p.E831 *	Nonsense	3	P	CF-causing
p.E92K	Missense	3	P	CF-causing
p.I1234V	Missense	3	P	CF-causing
p.I853I	Synonymous	3	VUS	N/A
p.L183I	Missense	3	P	N/A
p.M1101R	Missense	3	P	CF-causing
p.Q353 *	Nonsense	3	P	N/A
p.R334W	Missense	3	P	CF-causing
p.R352Q	Missense	3	VUS	CF-causing
p.S955A	Missense	3	VUS	N/A
p.V1198M	Missense	3	LP	N/A
c.1766 + 3A > G	Intronic	2	P	CF-causing
c.2491 − 51T > C	Intronic	2	VUS	N/A
c.3468 + 52A > C	Intronic	2	VUS	N/A
p.C866T	Missense	2	LP	N/A
p.E528E	Missense	2	P	N/A
p.F834L	Missense	2	P	N/A
p.G1069R	Missense	2	P	Varying clinical consequence
p.I1000fs*2	Frameshift	2	P	N/A
p.I1295fs *33	Frameshift	2	P	N/A
p.I752S	Missense	2	VUS	N/A
p.K64E	Missense	2	P	N/A
p.L137fs*15	Frameshift	2	P	N/A
p.M952T	Missense	2	P	Unknown significance
p.R709 *	Nonsense	2	P	CF-causing
p.R74W	Missense	2	P	Varying clinical consequence
p.S1373I	Missense	2	LP	N/A
p.T1019A	Missense	2	VUS	N/A
p.T1057A	Missense	2	P	N/A
p.T1299T	Missense	2	VUS	N/A
p.T966M	Missense	2	VUS	N/A
p.V201M	Missense	2	P	Unknown significance
p.V754M	Missense	2	P	Non CF-causing
p.Y301C	Missense	2	VUS	N/A
p.Y577H	Missense	2	VUS	N/A
c.1116 + 57C > G	Intronic	1	VUS	N/A
c.164 + 9A > T	Intronic	1	VUS	N/A
c.1680 − 756C > T	Intronic	1	VUS	N/A
c.2490+5G > T	Intronic	1	VUS	N/A
c.2909 − 15T > G	Intronic	1	VUS	N/A
c.2989 − 3C>T	Intronic	1	VUS	N/A
c.3139 + 80delA	Intronic	1	VUS	N/A
c.3368 − 4A > G	Intronic	1	P	N/A
c.3468 + 137T > C	Intronic	1	VUS	N/A
c.3469 − 2A > G	Intronic	1	P	N/A
c.3963+15T > C	Intronic	1	VUS	N/A
c.490 − 165T > C	Intronic	1	VUS	N/A
c.53+28A > G	Intronic	1	VUS	N/A
c.870 − 1026delC	Intronic	1	VUS	N/A
p.A1009T	Missense	1	P	N/A
p.A1113V	Missense	1	VUS	N/A
p.A1364A	Synonymous	1	P	N/A
p.A455V	Missense	1	P	N/A
p.D58G	Missense	1	P	N/A
p.D891G	Missense	1	P	N/A
p.D924N	Missense	1	P	Unknown significance
p.D985E	Missense	1	LP	N/A
p.E1104G	Missense	1	LP	N/A
p.E1409K	Missense	1	P	N/A
p.E826K	Missense	1	P	N/A
p.F508C	Missense	1	P	Non CF-causing
p.F994C	Missense	1	P	N/A
p.F994L	Missense	1	VUS	N/A
p.G314A	Missense	1	LP	N/A
p.G406A	Missense	1	VUS	N/A
p.G723D	Missense	1	LP	N/A
p.I125T	Missense	1	P	N/A
p.I521F	Missense	1	P	N/A
p.K1060T	Missense	1	P	N/A
p.K536E	Missense	1	LP	N/A
p.K612N	Missense	1	LP	N/A
p.K68N	Missense	1	VUS	N/A
p.L1156F	Missense	1	VUS	N/A
p.L227R	Missense	1	P	CF-causing
p.L233P	Missense	1	LP	N/A
p.L467F	Missense	1	P	N/A
p.L568F	Missense	1	VUS	N/A
p.L610I	Missense	1	P	N/A
p.L88 *	Nonsense	1	P	CF-causing
p.M1407T	Missense	1	P	N/A
p.N1432K	Missense	1	P	N/A
p.N306S	Missense	1	VUS	N/A
p.N417K	Missense	1	VUS	N/A
p.P111P	Synonymous	1	VUS	N/A
p.P5L	Missense	1	P	Varying clinical consequence
p.Q685R	Missense	1	LP	N/A
p.R1162L	Missense	1	P	Non CF-causing
p.R1438fs*10	Frameshift	1	LP	N/A
p.R31C	Missense	1	P	Non CF-causing
p.R334Q	Missense	1	P	Varying clinical consequence
p.R347H	Missense	1	P	CF-causing
p.R3W	Missense	1	VUS	N/A
p.R668L	Missense	1	P	N/A
p.R785 *	Nonsense	1	P	CF-causing
p.R997F	Missense	1	P	N/A
p.S1426P	Missense	1	P	N/A
p.S307N	Missense	1	P	N/A
p.S912L	Missense	1	P	Unknown significance
p.S945L	Missense	1	P	CF-causing
p.T1057R	Missense	1	LP	N/A
p.T351S	Missense	1	P	N/A
p.T465N	Missense	1	P	N/A
p.T760M	Missense	1	P	N/A
p.V855I	Missense	1	VUS	N/A
p.V920L	Missense	1	P	N/A
p.V938G	Missense	1	LP	N/A
p.W1098L	Missense	1	P	N/A
p.Y424Y	Synonymous	1	P	N/A
p.Y919C	Missense	1	P	N/A

P: Pathogenic, LP: Likely Pathogenic, VUS: Variant of Uncertain Significance, N/A: not available.

## Data Availability

The datasets used and/or analysed during the current study are avalible from the corresponding author upon reasonable request.
